# Knowledge of Genetic Counseling Among Patients With Breast Cancer and Their Relatives at a Nigerian Teaching Hospital

**DOI:** 10.1200/JGO.17.00158

**Published:** 2018-07-06

**Authors:** Prisca Adejumo, Toyin Aniagwu, Abimbola Oluwatosin, Omolara Fagbenle, Olubunmi Ajayi, Dasola Ogungbade, Adeyoola Oluwamotemi, Funmilola Olatoye-Wahab, Abiodun Oni, Oluyemi Olajide, Babatunde Adedokun, Temidayo Ogundiran, Olufunmilayo Olopade

**Affiliations:** **Prisca Adejumo**, **Abimbola Oluwatosin**, **Babatunde Adedokun**, College of Medicine, University of Ibadan, Ibadan; **Toyin Aniagwu, Omolara Fagbenle**, **Olubunmi Ajayi**, **Dasola Ogungbade**, **Adeyoola Oluwamotemi**, **Funmilola Olatoye-Wahab**, **Abiodun Oni**, **Oluyemi Olajide**, and **Temidayo Ogundiran**, University College Hospital, Oyo, Nigeria; and **Olufunmilayo Olopade**, The University of Chicago, Chicago, IL.

## Abstract

Breast cancer prevalence continues to increase globally, and a significant proportion of the disease has been linked to genetic susceptibility. As we enter the era of precision medicine, genetics knowledge and skills are increasingly essential for achieving optimal cancer prevention and care. However, in Nigeria, patients with breast cancer and their relatives are less knowledgeable about genetic susceptibility to chronic diseases. This pilot study collected qualitative data during in-depth interviews with 21 participants. Of these, 19 participants were patients with breast cancer and two were relatives of patients with breast cancer. Participants were asked questions regarding their knowledge of breast cancer, views on heredity and breast cancer, and views on genetic counseling. Participants’ family histories were used as a basis with which to assess their hereditary risk of breast cancer. Participant responses were audio recorded and transcribed manually. The study evaluated patients’ and relatives’ knowledge of genetic counseling and the use of family history for the assessment of familial risk of breast cancer. This will serve as a guide to the processes of establishing a cancer risk assessment clinic.

## INTRODUCTION

Breast cancer is the leading cause of cancer mortality among women in developing countries.^1,2^ In Nigeria, in particular, breast cancer is currently both the most common cancer among women and the leading cause of cancer mortality.^3-5^

Knowledge of genetics has become an increasingly important tool in cancer care services in many parts of the world.^6,7^ In recent years, it has been determined that as many as 5% to 10% of breast cancer cases can be attributed to inherited breast cancer susceptibility genes,^8,9^ with BRCA1 and *BRCA2* implicated as two key genes that have been associated with hereditary breast cancer susceptibility.^10,11^ With the availability of numerous high-throughput sequencing approaches to cancer screening, prognosis, and treatment, the prediction of treatment response has been revolutionized.^12^ As such, it is now possible that women with a high personal or familial risk for breast and ovarian cancer can obtain genetics-based risk estimations for breast and ovarian cancer by testing for the *BRCA1/2* mutation.^11^

It has become evident that breast cancer and its underlying genetic mechanisms vary by population.^13^ Nigerian patients with breast cancer, in particular, have an exceptionally high frequency of *BRCA1* and *BRCA2* mutations (7.1% and 3.9%, respectively),^14^ but it is currently unknown whether these patients recognize the potential issues that surround breast cancer genetics and heredity. Nigerian patients with breast cancer and their relatives may also not understand the function of breast cancer–associated genes, as they may not be familiar with the genetic implications or outcomes of their disease.^15^ As the dissemination of genetic findings and the clinical implementation of new genetic knowledge is key for translational research to achieve its full potential, it is crucial to be cognizant of patients’ current knowledge and understanding of cancer genetics and risk before creating widespread genetic counseling programs with which to address cancer risk and prevention.

The main purpose of genetic counseling is to help patients and their family members understand genetic diseases and risk, discuss disease and risk management options, and explain the risks and benefits of genetic testing.^16^ Counseling sessions focus on providing patients with vital, unbiased information and nondirective assistance during the patient’s decision-making process^17^; however, there is little information on how many and which women would want to address the possible genetic component of their breast cancer and take preventive measures at a time when their primary concern is survival.^18^ To this effect, it is crucial to gauge the knowledge of Nigerian patients with breast cancer and their relatives of genetic susceptibility and genetic counseling to better tailor genetic counseling programs and educational materials to their individual needs.

The current study sought to elicit patient and family members’ knowledge and attitudes about genetic counseling and the use of family history in assessing familial risk of breast cancer. In the process of assessing participants’ knowledge of breast cancer and heredity via the creation and use of family pedigrees, this study also sought to elicit participants’ concerns about genetic counseling.

## METHODS

This study relied on qualitative data from 21 in-depth interviews that were conducted at University College Hospital (UCH; Ibadan, Nigeria) from February 2015 to May 2015. Qualitative data were collected using in-depth interviews from 19 female adults with breast cancer who reported positive family history and two of their first-degree relatives (FDRs) from four oncology units of UCH Ibadan. A convenience sampling technique was used to select patients with breast cancer from four clinics or departments at UCH—the Surgical Outpatient Clinic, Radiation Oncology Clinic, Radiology Department, and Surgical Oncology Ward. Patients with a confirmed diagnosis of breast cancer history as well as their FDRs were approached by nurses trained in genetic counseling. Those who confirmed a positive family history of any cancer and gave informed consent were selected for a precounseling in-depth interview. In total, 19 adults with a self-reported family history of cancer were selected from a list of 102 patients, and two consenting FDRs of those patients also participated. Some demographic characteristics were obtained from participants.

Participants were asked questions about their knowledge of breast cancer, views about its hereditary causation, and views on genetic counseling and the use of family history to assess the hereditary risk of breast cancer. All questions were open ended and asked in a way that allowed patients and relatives to elaborate on topics that were seen as most important to them. Responses were recorded verbatim and transcribed manually.

This study was part of a larger study, titled “Effects of Genetic Nursing Education on Nurses’ Competencies in Counselling Cancer Patients and their Relatives in Selected Teaching Hospitals in Southwest Nigeria.” Ethical review committees of selected teaching hospitals approved the larger study.

## RESULTS

Twenty-one participants—19 patients with breast cancer and two FDRs—were interviewed. Transcripts and notes were manually analyzed to raise themes and identify emergent issues related to knowledge about cancer and breast cancer, pedigree, knowledge about genetic counseling, and concerns about genetics, and the process of genetic counseling.

Demographic characteristics demonstrated that more than one half (58%) of participants were within the age range of 40 to 59 years, and all participants were females. They were largely of Yoruba ethnicity (Table 1).

**Table 1:**
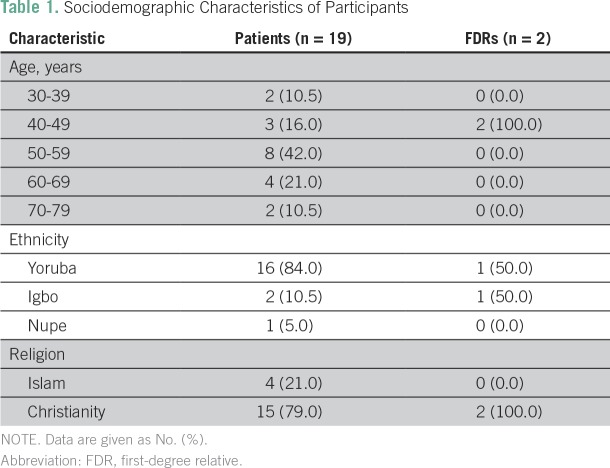
Sociodemographic Characteristics of Participants

**Fig 1:**
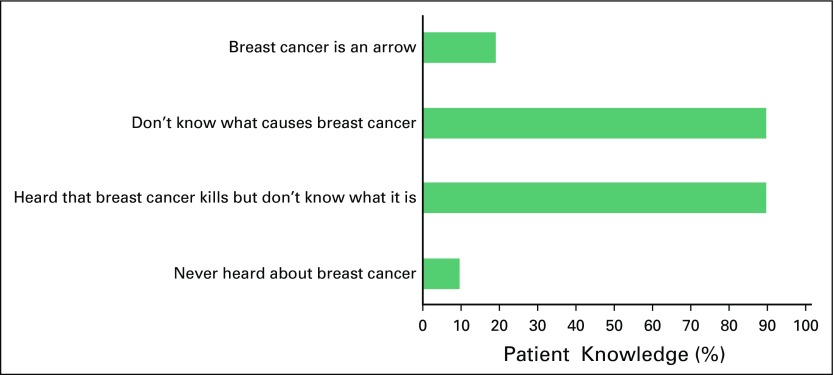
What participants know about breast cancer (N = 21).

The majority of participants (90.0%) claimed to have heard about breast cancer but did not have an idea of what it is exactly and what causes it (Fig 1). Some of the responses included:

I never heard about breast cancer until lately. I have no knowledge, though, I have seen some posters but I don’t understand them. I have no idea of its cause or causes.

*I heard that breast cancer kills although nobody ever told me how it happens or what causes it.* [This is the most prominent response from almost all respondents.]

I felt a small lump in my breast and went to a medicine store who directed me to general hospital where I had biopsy and they said it is cancer.

Approximately one fifth (19.0%) of participants perceived breast cancer to be a spiritual attack and called it an arrow (meaning a spiritual missile). According to one middle-aged female respondent, *“*I think my own cancer is an arrow. I went to a specialist private hospital and I was asked to come back for the test. My mother was sick with something like what I have now… ‘*Won ta won lofa ni*’” [she had a spiritual arrow sent to her]. Another female respondent stated that she went to various hospitals but they did not find anything, “I thought mine is spiritual too.”

The remaining 10% of participants stated that they had never heard about breast cancer before. Some of their verbatim responses included:

What breast cancer is or what causes it, I don’t know.

*I have never heard anything about breast cancer. Se ko ni ‘affect’ awon omo mi sa o?’* [I hope it will not affect my children in any way.]

**Fig 2:**
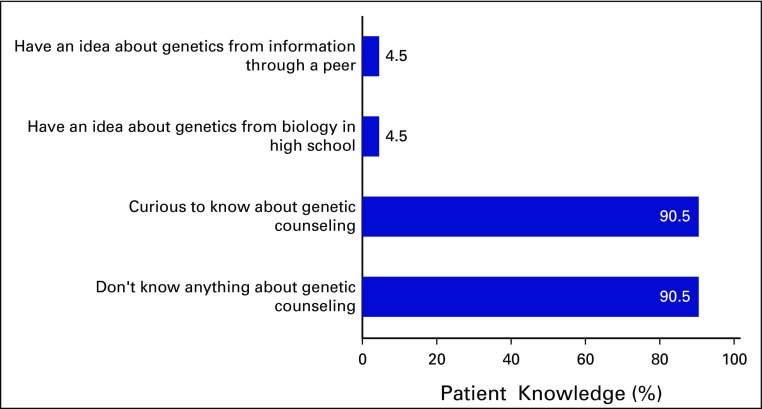
What participants know about genetic counseling (N = 21).

Similarly, 91% of participants did not know anything about the genetics of breast cancer or genetic counseling; these participants were curious to know what genetics is about (Fig 2). Some responses included:

I never heard about genetic counseling.

I never knew it is part of cancer care and management.

I trained here and I have nursed breast cancer patients, but I never gave them genetic counseling because I didn’t know about it and I will like to know.

We have come for treatment, not counseling. I don’t know anything about genetic counseling but it is a welcome idea.

I never had any information about cancer or genetic counseling.

…I also fear that my children may contract it from me.

I never heard any investigation called genetic testing for breast cancer, so I will like to have the counseling.

All participants, however, had concerns about genetic counseling. These concerns included participants’ inability to provide complete family history or being able to inform other family members about the possibility of having the disease (Table 2). Some of their verbatim responses reflected their concerns:

**Table 2:**
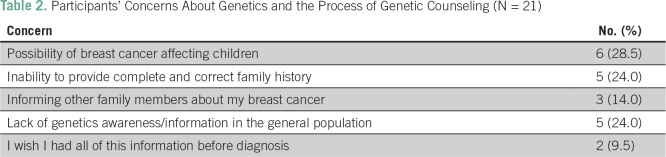
Participants’ Concerns About Genetics and the Process of Genetic Counseling (N = 21)

*To trace past generation is difficult, since they* [our parents] *did not tell us. How then are we supposed to know and provide accurate information here?*

Thank you for informing me about genetic information and counseling, but can one remember everything about one’s family?

Besides, to my mind, this is late, why didn’t you give us this information before we develop cancer? I never heard about breast cancer until about 3 years ago on radio during what they called ‘pink month’ celebration in University of Ibadan, then I said ‘God forbid.’ I don’t know how I can ever tell my sisters that I have cancer talk less of informing them about any test to find out the possibility of their own susceptibility, God forbid bad thing and come to think of it, why should I be the one to spread this type of bad news. This is tough.

My mummy’s pastor once told me that ‘it is not in your family, it will not start from you and she asked me to reject it.’ I am really afraid, I don’t want me or my children to have breast cancer.

## DISCUSSION

All participants in this study were females, which was not surprising considering it is the gender that breast cancer affects most. A majority of study participants reported that they had never heard about breast cancer or its causes, which is consistent with findings in the literature.^19,20^ In Nigeria, patients present in clinical settings late, likely as a result of their lack of knowledge about the disease.^21^ The findings of the current study also reveal that the majority of participants and their relatives who have heard about breast cancer said they were told “it is an arrow.” This belief has been variously reported among Nigerian women and it has been a factor in why Nigerian women present with advanced breast cancer. This notion deserves more pragmatic intervention to address it.^22-25^

In a study carried out in Botswana, patients with breast cancer were found to not have knowledge about the disease before diagnosis, which led to fear of the diagnosis, of death, and of misinterpretation of its signs and symptoms.^26^ These factors, combined with the influence of lay beliefs and advice from the community, led to a delay in seeking help at the hospital.^27^ A recent review of the literature of factors that contribute to the late presentation of patients with breast cancer in hospital also confirmed this result.^27^ The knowledge and beliefs of patients and their FDRs is important in their understanding of cancer genetics and potential genetic counseling. This has implications for improved health-seeking behavior and consequent increases in the uptake of screening services and early treatment. Addressing the lack of community knowledge and awareness immediately will likely go a long way in improving cancer care outcomes.

Almost all participants in the current study were curious to know about genetic counseling. They claimed to have never heard about it and were never before counseled in this regard. There is a dearth of literature on the knowledge of patients with breast cancer about genetic susceptibility and genetic counseling in Nigeria; however, in an American study of patients with breast cancer and FDRs of affected individuals, the population was moderately knowledgeable about breast cancer genetics, with an average score of 5.35 of 10.^28^ In another randomized controlled study of genetic testing for breast and ovarian cancer susceptibility in diverse groups of women in western Washington in the United States, it was found that a majority of women from each group had read or heard nothing or relatively little about genetic testing for breast cancer risk.^16^ Of particular note, African American women were least likely to have heard about such genetic testing during the study. In addition, despite this self-reported lack of knowledge of genetic testing for breast cancer risk, a large proportion of women from all study groups responded that they would be appropriate candidates for this genetic testing given their family history.^16^ In a similar study of female patients who awaited appointments at a large primary care clinic of the Group Health Cooperative (Seattle, WA), 47% of women had read or heard almost nothing about genetic susceptibility testing, and most did not know the answers to questions that assessed knowledge of breast cancer genetics.^29^ We found similarities to these studies in the Nigerian setting in which a larger proportion of patients did not know anything about genetic susceptibility to breast cancer or what genetic susceptibility might imply for them. This may be connected with the negative belief systems surrounding not inheriting what are thought to be bad genes from parents.

In Nigeria, information about genetics from health professionals was lacking until 1986, when genetic counseling in sickle-cell disease began in Lagos,^30^ and the situation has been worse for genetics and genetic counseling in Nigeria than in other countries as there had not been any resources for communicating the role genetics plays in cancer causation. Recent training conducted for nurses in teaching hospitals in southwestern Nigeria concluded that cancer genetics and genetic counseling is presently missing in the curricula of nursing programs and from comprehensive cancer care in Nigeria.^31^ The establishment of a cancer risk assessment program therefore becomes important for bridging the knowledge gap about cancer genetics between health professionals and patients.^17^

Some participants had concerns about the possibility of breast cancer affecting their children. Health education should address the misconception about cancer being contagious. Such misconceptions could create fear in family members and affect the necessary level of support needed by patients from their family members. Other concerns that have been raised by participants include how they can give a correct history if their parents did not provide them any information, as well as how they can inform their family members of their disease and their relatives’ susceptibility. The extent of secrecy in disclosing cancer diagnoses to others as well as social factors that influence the perception of familial cancers in Nigeria is reflected in these concerns and in the fact that people living with breast cancer are often stigmatized.^17,32^

In general, knowledge of genetics and genetic counseling was not adequate for patients with breast cancer and FDRs to understand the possible reason for their disease and its influence on prevention for others in their families. Despite the increasing body of evidence for the contribution of genetics to health or illness and genetic counseling, patients with cancer and their relatives in the current study do not know that this information is available; however, the participants welcome the innovation of genetic counseling and yearn to know more. It is clear that there is fertile ground for the application of genetics to the prevention and care of breast cancer in Nigeria. There is a pressing need to pilot comprehensive cancer care centers in Nigeria, which includes creating a cancer risk and prevention clinic for specific genetic services. Nonetheless, it is important that genetic counselors do not underestimate the existing knowledge that patients with breast cancer have concerning genetics and its importance in assessing genetic factors, because this knowledge influences the kind of genetic information given during counseling and the way it is received by patients and relatives.

There are various strengths and limitations in this study. This study was limited to a small number of female participants at one specific teaching hospital in Ibadan, Nigeria, which may limit the generalizability of its findings to other locations in Nigeria and to the knowledge and views of males with regard to cancer genetics, heredity, and genetic counseling. Moreover, only two of those participants were FDRs of the patients. Many patients and their relatives were anxious about the cancer diagnosis and apprehensive about treatments, so they felt that being interviewed was a waste of time; however, by conducting in-depth interviews, we were able to solicit more complete information and views from participants.

Despite an increasing body of evidence for the contribution of genetics to health and illness, the availability of information about cancer generally and about genetic counseling is still unknown to an overwhelming majority of patients with cancer and their FDRs in Nigeria; however, patients and their relatives expressed a willingness to receive information on cancer genetics as well as genetics counseling services if and when they were to become available. Having explored patients’ knowledge of and interests in cancer genetics and genetics counseling as a potential risk-prevention strategy in Nigeria, the findings of the current study indicate that the creation of a cancer risk and prevention clinic is a welcome idea in Nigeria.
